# Evaluating producer preferences and information processing strategies for drought risk management tools in Bangladesh

**DOI:** 10.1016/j.wdp.2019.100132

**Published:** 2019-09

**Authors:** David L. Ortega, Patrick S. Ward, Vincenzina Caputo

**Affiliations:** a Michigan State University, United States; b Duke-Kunshan University, China

**Keywords:** Equality constrained latent class model, Attribute non-attendance, Discrete choice experiment, Insurance, Drought-tolerant rice, Bangladesh

## Abstract

We use stated preference data to study farmers’ preferences for two alternative strategies to manage drought risks in Bangladesh. A choice experiment on drought tolerant rice varieties and weather index insurance was administered to 2306 farmers. Two overarching preference classes or meso-groups were identified using an equality constrained latent class model, each containing various patterns of attribute non-attendance. Our analysis finds that farmers rely on information processing strategies to simplify risk management tool decisions to mitigate drought risk. Differences in the use of farm inputs, and access to savings mechanisms, both formal and informal, are shown to affect decision making.

## 1 Introduction

For agricultural development projects to be successful, they need to meet the demand of the local context. In order to do so, researchers and practitioners need a better understanding of stakeholder preferences and their surrounding environment (Andersson and D'Souza, 2014, Corbeels et al., 2014). As such, the sustainability of development projects depends on their constituents’ ability to convey and articulate their needs. This is particularly important in situations where a novel product or technology is introduced.

Choice experiments (CE) have become an increasingly important tool used to study stakeholder preferences and behavior in developing countries.^1^ The scope of agricultural development topics that can be addressed using CEs and the heterogeneity by which agricultural and food policy issues can be informed with this method has been highlighted in recent studies (Bello and Abdulai, 2016, Ochieng et al., 2017, Ortega et al., 2016, Waldman et al., 2017, Ward et al., 2016, Ward et al., 2014). For example, Ward et al. (2014) employ a CE to assess farmer preferences for drought tolerant (DT) rice in India. They find that farmers value reductions in yield variability from DT varieties and are willing to pay for seeds that offer yield advantages under normal conditions. Similarly, Ortega et al. (2016) use a CE to examine farmer’ preferences for various legume-maize intercrop systems in Malawi and find that farmers significantly discount legume yields in favor of maize yields despite the additional benefits provided by legumes. Ochieng et al. (2017) utilize a CE to analyze farmer preference heterogeneity for supermarket contract attributes to better inform design and enhance farmer participation in supermarket procurement channels in Kenya.

One of the most undervalued advantages of CEs in agricultural development applications is the ability to assess and evaluate behavior or policies ex ante. Given that most assessment studies are conducted ex post, or after a sufficient time lapse to allow for the gathering and analysis of behavioral data, CEs can be implemented at the onset of a program to better inform project design (Knowler, 2015).

Farmers in developing countries face a great deal of production risk. This is specially the case for rice farmers in Bangladesh who face a constant threat of extreme weather events. While Bangladesh is most often associated with floods and cyclones given its subtropical monsoon climate, the country has over 5 million hectares of drought-prone areas. As a result of climate change, Bangladesh’s exposure to weather risk, including both droughts and floods is likely to increase (Nelson, 2009). Drought intensity, specifically, is predicted to increase in various climate change scenarios, significantly increasing the regions prone to severe drought (Rahman et al., 2007).

Financial and technological innovations that mitigate weather-related production risks have the potential to greatly benefit farmers in many risk-prone areas. Typically, households often rely on informal mechanisms such as informal savings, gifts, and loans to cope with various types of risks (Alderman and Paxson, 1992, Clarke et al., 2012, Dercon, 1996, Dercon et al., 2006, Fafchamps and Lund, 2003). The ineffectiveness of these strategies highlights the need for more formal mechanisms to mitigate these risks (Santos, Sharif, Rahman, & Zaman, 2011). In Bangladesh, drought-tolerant (DT) rice varieties and weather index insurance (WII) have been proposed as two alternative strategies to help farmers manage risks associated with drought. The former is a technological innovation that reduces the impact of drought stress on rice production, sequestering an important component of farmer livelihoods from the adverse effects of droughts. The latter is a financial innovation that allows farmers to shift a portion of their risk to the insurer, who compensates them in the event that a certain level of drought stress occurs. Recent work on farmer demand illustrates how these tools can independently address drought risk and demonstrates the additional benefits gained by combining them in a complementary risk management product (Ward and Makhija, 2018, Ward et al., accepted for publication).

Understanding farmer preferences and demand for these risk management tools is critical for the development of specific rice varieties and insurance packages that meet the needs of farmers. This need is highlighted given that assessment studies of these types of technologies require a significant monetary investments and sufficient time, usually covering multiple growing seasons, to measure uptake. Moreover, this type of information (priors on preferences) could be used to test specific rice varieties and insurance package designs that align more closely with the local needs and farmer preferences. As such, choice experiments serve as a tool that can be used to learn about farmer’s information processing strategy to better develop and target information for managing drought risk.

This study builds on a previous investigation on farmer demand for financial and technological tools for managing drought risk (Ward et al., accepted for publication) and investigates how farmers rely on alternative strategies to process information and simplify choice decisions. In traditional stated choice studies, it is assumed that respondents consider all attributes when assessing various choice alternatives. However, recent work suggests that individuals may not actually attend to all attributes when formulating their choices, and accounting for this behavior is important for deriving more accurate choice predictions and welfare estimates (Caputo et al., 2013, Scarpa et al., 2013). This is likely to be relevant in developing country studies, since study participants (e.g. farmers) often have low levels of education and incomes, and poverty is known to impede cognitive function, placing real limits on an individual’s decision making ability (Mani et al., 2013, Mullainathan and Shafir, 2013, Ortega and Ward, 2016).

There are two main approaches used to capture attribute non-attendance (ANA) behavior: The stated approach directly asks respondents whether or not they ignored attributes when making choices (e.g. Alemu et al., 2013, Carlsson et al., 2010, Hensher et al., 2005, Scarpa et al., 2010), and the inferred approach relies on analytical modeling techniques rather that individual’s self-assessment (e.g. Campbell et al., 2011, Hess and Hensher, 2010, Scarpa et al., 2009). Studies comparing these two methods have found discrepancies on which method to undertake (Caputo et al., 2018, Kragt, 2013, Mariel et al., 2013, Scarpa et al., 2010, Scarpa et al., 2013). Kragt (2013) finds little concordance between stated and inferred ANA, suggesting that respondents may not make attendance statements truthfully, calling into question the validity of the stated ANA approach. This body of literature has mainly focused on studying and modeling attribute non-attendance (ANA) behavior of respondents in developed country applications. Research addressing ANA behavior in developing countries is starting to emerge, with Ortega and Ward, 2016, Bello and Abdulai, 2016 being some pioneer studies in this area.

In this study, we use data on rice farmers’ preferences for two alternative strategies to manage drought risks in rural Bangladesh: drought-tolerant (DT) rice varieties and weather index insurance (WII). We administered a choice experiment to 2306 farmers and employ an equality constrained latent class (ECLC) model specification to capture both preference heterogeneity and ANA behavior. We utilize class membership probabilities and covariates to identify and describe farmers exhibiting differing information processing strategies. The remainder of this study is organized as follows: in the next section we describe our study area, data and choice experiment design. We then introduce the ECLC model and describe its flexibility in capturing ANA behavior (Section 3). Section 4 presents our empirical results and describes characteristics of farmers exhibiting different patterns of ANA behavior. Section 5 provides a discussion of how our findings can inform development interventions, and Section 6 concludes the study with a summary of our findings.

## 2 Data sources and experimental setting

**Fig. 1:**
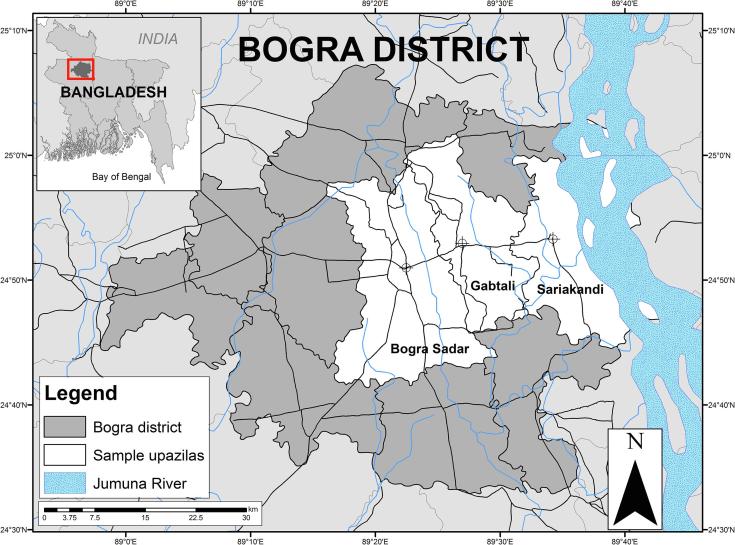
Sample area: Bogra, Gabtali, and Sariakandi Upazilas, Bogra district, Rajshahi division, Bangladesh.

The data used in this study come from a discrete choice experiment implemented as part of a midline survey that itself is part of a larger evaluation of agricultural risk management in Bangladesh. The survey was conducted during May and June 2014 in three *upazilas* (subdistricts) of Bogra district, in Rajshahi division in northwestern Bangladesh (Fig. 1).^2^ In the first year of the evaluation (2013), an index insurance product was marketed to members of a local Nongovernmental Organization (NGO), Gram Unnayan Karma (GUK). GUK primarily operates as a microfinance institution through its network of village-level women’s self-help groups. Prior to the initiation of the 2013 index insurance pilot, 40 villages were randomly selected from each of the three *upazilas,* for a total of 120 villages. Households in 60 randomly selected villages were offered weather index insurance, while households in the other 60 were not. Within each of the 120 villages, 20 households (on average) were randomly selected from GUK’s rosters. Because some households had either moved or could not be located, we were not able to interview all of the households that had participated in the baseline survey conducted during 2013. Our ultimate sample consisted of 2306 households across the three *upazilas*.

The choice experiment was designed to reflect agricultural risk management decisions that farmers in Bangladesh might face when considering strategies to mitigate drought risk. The attributes incorporated in the choice experiment are summarized in Table 1, but we describe them here in greater detail. The first attribute included in the choice experiment was constructed to reflect the inherent drought-tolerance of the rice variety being cultivated. Similar to Ward et al. (2014) and Arora, Bansal, and Ward (2019), this drought-tolerance was essentially presented as a series of discrete yield distributions; in other words, rather than simply considering the variety’s expected yield, respondents were asked to consider the variety’s potential yields under different weather conditions (normal conditions, moderate drought stress, and severe drought stress). This attribute was coded as a binary variable, with one variety reflecting the yield distribution of BRRI dhan 56, a risk reducing variety, and the other reflecting the yield distribution of BR 11, the most commonly grown high yielding *aman* rice variety.^3^

**Table 1:** Summary of choice experiment attributes and corresponding levels.

Potential yields under various weather conditions (t ha^−1^)	Level	Variety	Normal conditions	Moderate drought stress	Severe drought stress	Extreme drought stress
	1	Non-DT	6.5	2.6	1.0	0.3
	2	DT1 (BRRI dhan 56)	5.2	3.2	1.6	0.3

Duration (days from nursery to harvest)	Level	Label	Days			
	1	Short	<120			
	2	Medium	120–135			
	3	Long	>135			

Weather index insurance	Level	Label	Trigger (consecutive dry days)	Insurance payment (BDT)		
	1	No insurance				
	2	Full coverage, low trigger	12	300		
	3	Full coverage, high trigger	14	600		
	4	Limited coverage, low trigger	14	300		
	5	Limited coverage, high trigger	18	600		

Bundle price	Level	Bundle price (BDT)				
	1	20				
	2	60				
	3	80				
	4	120				
	5	150				

Notes: DT refers to drought tolerant rice variety (in this case, BRRI dhan 56), and 1 Bangladeshi Taka (BDT) = 0.0129 USD at the time of the study.

**Table 2:** Equality constrained latent class model estimates.

	Meso-group 2								Meso-group 2					
	Class 1: AA1			Class 2: ANA		Class 3: ANA – Seed			Class 4: AA2			Class 5: ANA – Financial		
Utility coefficient	Coeff.		S.E.	Coeff.	S.E.	Coeff.		S.E.	Coeff.		S.E.	Coeff.		S.E.
Drought tolerance (DT)	−6.446	***	1.072	0	NA	0		NA	−0.492	***	0.058	−0.492	**	0.058
Short duration	6.582	***	1.199	0	NA	0		NA	0.970	***	0.074	0.970	***	0.074
Medium duration	2.266	***	0.394	0	NA	0		NA	0.383	***	0.057	0.383	***	0.057
Limited coverage, low trigger	4.375	***	0.866	0	NA	4.375	***	0.866	1.683	***	0.170	0		NA
Limited coverage, high trigger	6.565	***	1.210	0	NA	6.565	***	1.210	1.887	***	0.187	0		NA
Full coverage, low trigger	2.859	***	0.388	0	NA	2.859	***	0.388	2.102	***	0.164	0		NA
Full coverage, high trigger	5.823	***	0.997	0	NA	5.823	***	0.997	2.720	***	0.189	0		NA
Price (negative, 100 Taka)	−0.011	***	0.002	0	NA	−0.011	***	0.002	−0.010	***	0.001	0		NA
ASC – Status Quo	−4.310	***	0.728	0	NA	−4.310	***	0.728	−3.699	***	0.237	−3.699	***	0.237
Probability of class membership	0.360			0.010		0.033			0.434			0.163		
Number of households (n)				2306										
Number of choice sets per household (J)				5										
Total number of observations (N)				11,530										
Parameters estimated (K)				22										
Log-likelihood				−6214.20										
Adjusted Pseudo-R^2^				0.5089										

Notes: *** indicates statistical significance with at most 1 percent chance of Type I error; ** indicates statistical significance with at most 5 percent chance of Type I error; * indicates statistical significance with at most 10 percent chance of Type I error. Models estimated using NLOGIT 5.0. Base level of the attributes include non-DT variety, long duration, and no insurance.

One of the other ways in which BRRI dhan 56 helps farmers to manage drought risk is through its short duration. The benefits of short duration are multifaceted: short duration allows for late transplanting in the case of delayed monsoon onset or, in the case of normal monsoon onset, the short duration allows for early harvesting, either to avoid late-season droughts, to cultivate a short-duration crop prior to the *boro* rice crop, or to begin preparing for the *boro* crop early. There is typically a trade-off, however, between duration and yield: shorter duration varieties almost universally yield less than longer duration varieties. Thus, while duration is not nearly as important (and hence valuable) as yields, Ward et al. (2014) demonstrated that within their sample, farmers did, on average, place a premium on medium and shorter durations, perhaps because of the reduced exposure to monsoon variability at either end of the typical season. For the purposes of our choice experiment, we allowed for three different levels of rice crop duration: short (less than 120 days), medium (120–135 days), and long (greater than 135 days).

The choice experiment also included an attribute related to weather index insurance. This attribute consisted of varying strike points (or triggers) and corresponding payments. This allowed us to isolate farmers’ valuations for each of these trigger/payment combinations. The insurance characteristics included in the choice experiment correspond to the trigger/payment combinations from a limited-coverage policy meant to complement the benefit profile of the drought-tolerant rice variety as well as a full-coverage policy that is essentially a standalone risk management product. Each of these insurance policies can be conceptualized as having two strike points, based on the most severe dry spell that was endured throughout the course of the monsoon season. The full coverage policy could be structured to provide compensation to farmers when there is at least a severe drought, with compensation roughly equivalent to the value of lost production. In this case, the full coverage policy might provide BDT^4^ 300 in compensation for a drought lasting 12–13 days, or BDT 600 for a drought lasting at least 14 days. The limited coverage policy, which, again, is meant to complement the performance profile of the drought-tolerant variety, would not need to compensate farmers under the same drought conditions as the full coverage policy, since the drought tolerant variety would be able to withstand some level of drought stress. Such a limited coverage policy might be structured to provide BDT 300 for droughts lasting 14–17 days, and BDT 600 for droughts lasting at least 18 days. In the choice experiment, we allowed for each of these trigger/payment combinations to be a separate attribute level. Thus, this attribute took a total of five levels: no insurance, a policy paying BDT 300 after a 12-day dry spell (“full coverage, low trigger”), a policy paying BDT 300 after a 14-day dry spell (“limited coverage, low trigger”), a policy paying BDT 600 after a 14-day dry spell (“full coverage, high trigger”), and a policy paying BDT 600 after an 18-day dry spell (“limited coverage, high trigger”).

As a final attribute, we included the full price for managing drought risk on 10 decimals of land (0.10 acres). The bundle price takes on a wide range of prices, since the farmer hypothetically could choose to purchase neither drought-tolerant rice nor insurance, could choose one or the other, or could choose to purchase both products. Including a wide range of prices forces the farmer to make difficult choices among the alternatives he or she is presented with, which in turn reveals information about the relative importance of different attributes in his or her utility function. Price levels included in the choice experiment were BDT 20, BDT 60, BDT 80, BDT 120, and BDT 150. The lower bound of this range is meant to roughly reflect the cost of purchasing seed from an informal dealer, while the upper bound of this range is meant to roughly reflect the cost of a bundled product including an actuarially-fair priced insurance product and certified drought-tolerant seed.

**Fig. 2:**
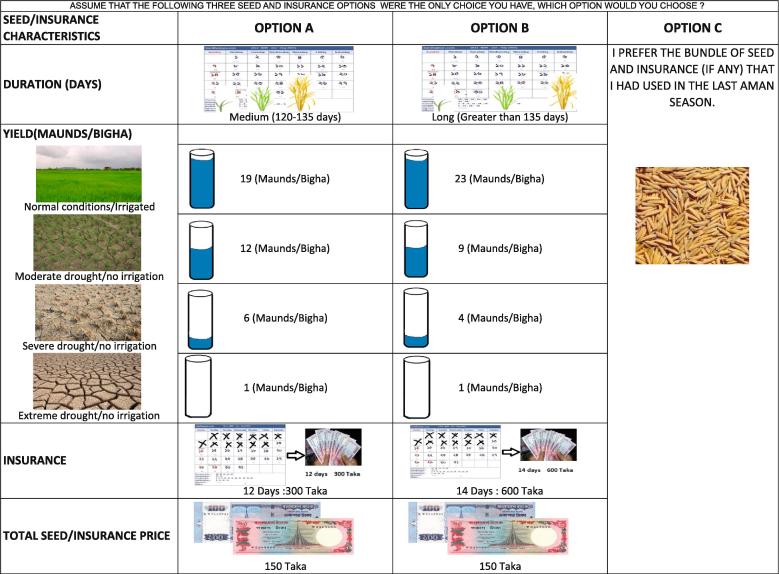
Example of illustrated choice card presented to survey participants. Notes: 1 Maund is approximately equal to 40 kg. 1 Bigha is approximately equal to 1/3 acre. 1 Bangladeshi Taka (BDT) = 0.0129 USD at the time of the study. Choice cards shown to study participants were translated into the local language (Bangla).

For the choice experiment, we included two hypothetical seed/insurance bundles as well as an option to revert to the rice variety cultivated during the 2013 *aman* season. We specified a D-optimal design using a modified Fedorov algorithm with a full-factorial candidate set, eliminating any candidate sets in which one option clearly dominated the other. From the design, we generated 30 unique choice tasks, which were then randomly allocated into six blocks, each comprised of five choice tasks. Households in the sample were then randomly allocated across the six blocks. While enumerators facilitated the choice experiment, the participants were given illustrated choice cards for each choice task to help participants better understand the different characteristics of the alternatives they were being asked to choose over. These illustrations were presented in Bangla, the primary language used throughout Bangladesh. An example of one of these illustrated choice cards is shown in Fig. 2.

## 3 Data analysis

The choice experiment responses were estimated using an ECLC model for panel data. The ECLC model is based on classes embedding different forms of attendance to attributes during the panel of observed choices (Campbell et al., 2011, Caputo et al., 2013, Hensher and Greene, 2010, Scarpa et al., 2009, Scarpa et al., 2013). Based on the assumption underlying individuals’ preferences, two different ECLC models can be specified: The first are ECLC models that allow individuals to belong to a certain latent class with a zero coefficient for certain attributes, while non-zero coefficients are assumed to be the same across the classes (Campbell et al., 2011, Scarpa et al., 2009). In these models classes differ on the basis of the particular pattern of zero-valued coefficients due to ANA behavior, but do not differ in terms of taste intensities (e.g. only one preference class), thereby ignoring preference heterogeneity. Second, there are ECLC models that account for the presence of separate classes of taste intensity as well as various patterns of attribute non-attendance (Caputo et al., 2013, Caputo et al., 2018). In this case the classes differ from each other in having both different values of taste intensities (e.g. multiple preference classes), and in having different subsets of attribute coefficients set to zero in accordance with different forms of ANA.

In this study farmers’ groups may differ not only in terms of patterns of attribute attendance, but also in terms of taste intensities. Capturing preference heterogeneity is particularly important in the present context given heterogeneity in farm household characteristics as well as levels of experience with using various risk mitigation strategies. Hence, an ECLC model accounting for both the presence of separate classes of taste intensity and various patterns of attribute attendance was estimated. Formally, in the ECLC model, the unconditional probability of the observed panel of *T* choices by respondent *i* is a weighted average over the *C* classes, with weight π*_ic_*, and each ANA class has indirect utility which embeds the zero-constrained coefficients:

(1) $$P_{\mathrm{ijt},c}=\sum_{c}\pi_{\mathrm{ic}}\prod_{t=1}^{T}\frac{exp({\beta_{c}^{\text{'}}x}_{\mathrm{ijt}|c})}{\sum_{g}exp\left(\beta_{c}^{\text{'}}x_{\mathrm{igt}|c}\right)}$$

where $\beta_{c}^{\text{'}}$ refers to the coefficients of the experimentally designed attributes, which can either attended or not attended depending on the attribute attendance assumptions underlying the different classes; $x_{\mathrm{ijt}}$ is a *k-*dimensional vector of observed experimentally designed attributes and their levels related to individual *i,* alternative *j,* and choice task *t.* These are represented by the attributes and attribute levels illustrated in Table 1: potential yields under various weather conditions (${\mathrm{DT}}_{\mathrm{ijt}})$, crop duration to harvest (${\mathrm{SD}}_{\mathrm{ijt}}$ and ${\mathrm{MD}}_{\mathrm{ijt}}$), weather index insurance ($LIM\_{\mathrm{LOW}}_{\mathrm{ijt}}$, ${LIM\_HIGH}_{\mathrm{ijt}},FULL\_{\mathrm{LOW}}_{\mathrm{ijt}}$, ${FULL\_HIGH}_{\mathrm{ijt}}$), bundle price (${\mathrm{PRICE}}_{\mathrm{ijt}}$), and a status quo constant (ASC_SQ). Given this experimental set up the general utility specification can be expressed as follows:

(2) $$U_{\mathrm{ijt}}=\beta_{1}{\mathrm{DT}}_{\mathrm{ijt}}+\beta_{2}{\mathrm{SD}}_{\mathrm{ijt}}+\beta_{3}{\mathrm{MD}}_{\mathrm{ijt}}+\beta_{4}{LIM\_LOW}_{\mathrm{ijt}}+\beta_{5}{LIM\_HIGH}_{\mathrm{ijt}}+\beta_{6}{FULL\_LOW}_{\mathrm{ijt}}+\beta_{7}{FULL\_HIGH}_{\mathrm{ijt}}+\beta_{8}{\mathrm{PRICE}}_{\mathrm{ijt}}+ASC\_SQ+\varepsilon_{\mathrm{ijt}}$$

The error term, $\varepsilon_{\mathrm{ijt}},$ is independent and identically distributed and follows a Gumbel (extreme value type I) distribution. With the exception of PRICE, which is treated as a continuous variable, the other experimentally designed attributes are dummy coded. The constraints on the coefficients of these attributes are dictated by different ANA patterns across classes.

## 4 Results

A model specification search, which included estimation of multinomial logit (MNL), random parameters logit (RPL), latent class (LC), and ECLC models, was conducted. We remind the readers that that 1) the MNL model implies homogeneity in farmers’ preferences; and 2) the RPL and LC models specify preference heterogeneity to occur continuously and discretely, respectively. Unlike the ECLC models discussed previously, the MNL, RPL and LC assume that respondents process or attend to all the information presented to them during the experiment.

Table A1 provides model information criteria for these models and corresponding utility specifications. The lower the information criterion value, the better the model fit. While it is well known that the Bayesian Information Criterion (BIC) and Akaike Information Criterion (AIC) tend to under- or over-fit models, respectively, Dias (2006) shows that the modified Akaike Information Criterion (3AIC) outperforms both BIC and AIC. Given our large sample size, AIC and 3AIC provide similar results. Our findings indicate improvements of model fit when incorporating ANA behavior (models 3–7), suggesting that ANA behavior is a pattern characterizing how farmers make choices in this context, and when allowing for preference heterogeneity (model 8 and 11).

Thus, we now turn our attention to the ECLC models. As can be also seen from Table A1, we estimated two sets of ECLC models. The first set refers to ECLC models (models 3–7) in which farmers have same preferences (taste intensity) for the examined attributes but different patterns of attribute attendance. In the second set of ECLC models (models 9–10), on the other hand, farmers differ in terms of both preferences (taste intensities) and patterns of attribute attendance. The optimal number of classes, and thus the selection of the best model, was determined based on 3AIC, and these criteria were also used to assess whether additional segments provide any further economic information, with the overall aim of attaining segment parsimony.

In this application, these criteria are minimized in a model considering two overarching preference classes, hereafter referred to as *meso-group* (model 10). These *meso-groups* represent two distinct classes of farmers with a unique set of preferences. Each *meso-group* is comprised of various sub-classes exhibiting different patterns of attribute attendance. Hence, *meso-groups* differ in terms of both preference intensity and attribute attendance patterns*.* To illustrate, *meso-group 1* (c = 1) includes three attribute attendance classes: one complete attendance class (i.e., a class in which all attributes are attended, AA1), one complete non-attendance or random choice class (i.e., a class in which no attributes are explicitly attended to, ANA), and one class in which a pair of attributes (incidentally both pertaining to the seed; namely the yield distribution and duration) are not attended to (ANA-Seed). The complete ANA class represents those individuals who are essentially making choices at random throughout the choice experiment. Accordingly, the coefficients of all experimentally designed attributes in these classes are set to zero. In the case of the ANA-Seed class, only the coefficients representing yield distribution and duration are set to zero, while the others are constrained to have the same coefficient values of the complete attendance class. *Meso-group 2* (c = 2) contains two attribute attendance classes: one complete attendance class (AA2), and one class in which a pair of attributes (in this case, both representing financial characteristics; namely the insurance trigger/payment components and the bundle price) are not attended to (ANA-Financial). Accordingly, in the latter class the coefficients of the insurance and bundle price attributes are set to zero, while the others are constrained to have the same coefficient values of the complete attendance class. Importantly, while members of AA1 and AA2 each attended to all of the product characteristics, we allow for the intensity of their preferences to differ, as in a standard latent class approach.

The results from estimating the ECLC model using maximum likelihood are reported in Table 2. *Meso-group 1*, represents approximately 40 percent of farmers (belonging to one of three attribute attendance classes: AA1, ANA, and ANA-Seed), while *meso-group 2* has an estimated membership of approximately 60%, shared across only two attribute attendance classes (AA2 and ANA-Financial). Overall, the results suggest that there is a high probability that individuals in the sample fully attended to all of the choice attributes, with nearly 80 percent of respondents belonging to the full attendance classes in both *meso-groups* (AA1 and AA2). In *meso-group 1*, results also indicate that the vast majority of participants likely took the exercise seriously, as only 1 percent of farmers responded in such a way as to be completely random (ANA). Finally, among those classes where it is assumed pairs of attributes sharing similar characteristics to be ignored, there is a considerably greater likelihood that individuals fall into the class ignoring the financial characteristics (ANA-Financial in meso-group 2) than into the class ignoring the agronomic characteristics (ANA-Seed in meso-group 1).

Preference estimates across the two meso-groups are significantly different from zero and the pattern of signs and magnitudes demonstrates the consequences of our model specification. To illustrate, for those in AA1, ANA, and ANA-Seed classes, preferences for the insurance components and bundle price are the same, while preferences for seed characteristics are different, with members of AA1 having non-zero preferences, and those in Class ANA-Seed – ignoring these characteristics – having taste parameters equal to zero. Based on these preferences, we suggest that those farmers in *meso-group 1* (AA1, ANA, and ANA-Seed) generally dislike or ignore the seed characteristics – particularly the drought-tolerant yield distribution. This latter point is especially true amongst farmers in AA1, who exhibit a large and statistically significant disutility associated with this characteristic. This effect, we suggest, is due to the yield penalty for the drought tolerant variety under normal or well-irrigated conditions. While farmers in AA1 demonstrate some preference for short and medium duration vis-à-vis long duration (perhaps due to the expanded opportunities for increasing net sown area over the course of the year), farmers in ANA-Seed ignore the duration as well as the yield distribution (as do those in ANA, since they are assumed to be making choices completely at random). Within meso-group 2, members have identical preferences for the seed characteristics, but since members of ANA-Financial ignore the insurance and bundle cost characteristics, their taste coefficients for these attributes are zero, while coefficients for these attributes among members of Class AA2 are non-zero. While it would not be fair to say that those in AA2 dislike the insurance components, since these attributes generate positive utility, the relative weakness of these preferences is indicative that these components are only marginally important in the decision making process.

Because the taste coefficients reported in Table 2 are not the ‘true’ taste coefficients corresponding to each of the different classes, and the ‘true’ taste coefficients are scaled by an unobservable scale parameter which may vary across classes, it is not possible to make direct comparisons in preferences across groups based solely on the estimated utility coefficients (e.g., Burke et al., 2010, Swait and Louviere, 1993). The typical approach in such cases is to convert the estimated utility coefficients into willingness-to-pay by taking the ratio of the marginal utility of an attribute to the marginal utility of income, which is assumed to be equivalent to the marginal disutility of cost or price.

These estimates of demand show that farmers view these financial and technological tools as complementary for managing drought risk (Ward et al., accepted for publication). Our approach here, however, focuses on comparing the household characteristics of the farmers most likely to fall into each of these classes in an attempt to identify household, farm, or other socioeconomic characteristics that may be underlying preferences which are useful for targeting agricultural risk management interventions. To determine an individual respondent’s class membership, we consider each individual’s probability of belonging to one of the two *meso-groups*, differing in terms of taste intensity and ANA patterns (agronomic vs. financial characteristics).^5^

We report descriptive statistics for a series of household, farm, and financial characteristics across these two meso-groups in Table 3. Columns (1) and (2) report means or frequencies for a series of characteristics for meso*-*group *1* and meso*-*group *2*, respectively. In column (3), we report F-statistics from one-way analysis of variance (ANOVA) used for testing whether meso*-*group means in continuous variables differ, while column (4) report Pearson’s $\chi^{2}$ statistics for testing the hypothesis that the observed differences in the proportional frequency of categorical variables arose simply due to chance.

**Table 3:** Characteristics of households from different aggregated latent classes.

		(1)	(2)	(3)	(4)
		Meso-group 1: AA1 ANA ANA-Seed	Meso-group 2: AA2 ANA-Financial	ANOVA F-statistic (p-value)	Pearson χ^2^ statistic (p-value)
		n = 1072	n = 1234		
*Household characteristics*					
	Age of household head	43.05	41.50	1.78 (0.182)	
		[11.47]	[11.93]		
	Gender of household head (male = 1)	94.12%	92.22%		3.24 (0.07)
	Household head can neither read nor write	15.30%	15.96%		2.32 (0.51)
	Household head can sign only	36.47%	33.47%		
	Household head can read only	0.19%	0.16%		
	Household head can read and write	48.04%	50.41%		

*Farm characteristics*					
	Average rice yields across all plots (kg/decimal)	16.65 [7.88]	16.88 [7.23]	1.29 (0.26)	
	Total rice area planted across all plots (decimal)	57.48 [54.52]	55.43 [52.53]	0.76 (0.38)	
	Used urea (=1)	93.35%	93.50%		0.02 (0.89)
	Total expenditures on urea	1292.15	1225.06	1.15 (0.28)	
		[1463.68]	[1435.42]		
	Used TSP/SSP (=1)	70.97%	63.50%		14.45 (<0.001)
	Total expenditures on TSP/SSP	881.777	872.22	0.02 (0.89)	
		[1256.22]	[1337.84]		
	Used DAP (=1)	36.55%	32.60%		3.95 (0.05)
	Total expenditures on DAP	876.5578	667.502	7.47 (0.01)	
		[1336.95]	[736.97]		
	Used MoP (=1)	67.13%	60.00%		12.53 (<0.001)
	Total expenditures on MoP	628.95	554.04	3.05 (0.08)	
		[848.29]	[786.36]		
	Used pesticides (=1)	74.63%	70.41%		5.09 (0.02)
	Total expenditures on pesticides	488.468	445.9249	0.99 (0.32)	
		[861.74]	[878.91]		
	Used irrigation (=1)	72.85%	72.50%		0.03 (0.85)
	Total expenditures on irrigation	3277.879	3034.187	1.22 (0.27)	
		[5086.70]	[3914.85]		
	Used power tiller (=1)	93.08%	91.65%		1.65 (0.20)
	Total expenditures on power tiller use	1540.07	1460.639	1.25 (0.26)	
		[1624.89]	[1640.65]		

*Financial characteristics*					
	Any savings account with a bank (=1)	30.50%	27.39%		2.71 (0.10)
	Member of informal savings group (=1)	16.04%	23.50%		19.91 (<0.001)
	Has savings box in residence (=1)	30.97%	30.96%		0.0001 (0.99)
	Savings ability in a good month (Tk)	1281.10 [3905.63]	1620.89 [5952.56]	2.54 (0.11)	
	Household has more than enough savings	41.79%	48.95%		12.56 (0.01)
	Household has enough savings for regular consumption needs and small unexpected expenditures	23.13%	21.47%		
	Household has just enough savings for regular consumption needs	13.81%	11.51%		
	Household does not have enough savings for consumption needs	21.27%	18.07%		
	Adult household member has taken a loan in the last 12 months (=1)	93.19%	94.00%		0.63 (0.43)
	Ever purchased weather/crop insurance (=1)	13.49%	11.36%		2.38 (0.12)
	Friend/neighbor/relative ever purchased weather/crop insurance (=1)	15.66%	13.91%		1.38 (0.24)
	Purchased drought insurance in 2013	37.41%	38.01%		0.088 (0.767)
	Purchased drought insurance in 2013 if part of treatment group	39.83%	37.76%		0.50 (0.48)
	Units of drought insurance purchased in 2013	2.65 [2.94]	2.91 [3.41]	1.44 (0.23)	
	Units of drought insurance purchased in 2013 if part of treatment group	3.31 [3.63]	3.73 [4.12]	1.27 (0.35)	

*Geographic characteristics*					144.95 (<0.001)
	Bogra Sadar upazila (=1)	42.94%	31.62%		
	Gabtoli upazila (=1)	26.66%	34.04%		
	Sariakandi upazila (=1)	30.40%	34.34%		

Notes: In columns (1) and (2), standard deviations in brackets. In columns (3) and (4), p-values from F- and $\chi^{2}$ tests in parentheses.

In terms of characteristics of the respondent, there is not much of a difference between those in meso*-*group *1* and meso*-*group *2*. The age of the household head (who was the respondent in virtually all instances) is essentially the same in both groups, and there is no discernable difference in the literacy rates. There is a slight difference in the proportion of male decision makers, though since virtually all decision makers are male (more than 93 percent in the entire sample), it is difficult to articulate a compelling narrative to characterize this phenomenon.

Where some systematic differences in the two meso-groups do emerge is in regards to farm-level characteristics. While there are no systematic differences in rice yield or in rice area cultivated, there arise some significant differences in the proportion of farmers who use various inputs, particularly chemical inputs, with farmers in the first meso-group having a significantly higher proportion of farmers using almost all agro-chemicals. There is a significant difference in the proportion of farmers using various fertilizers, including triple and single super phosphate (TSP and SSP, respectively; 70.97 percent vs. 63.5 percent), diammonium phosphate (DAP; 36.55 percent vs. 32.6 percent), and muriate of potash (MoP; 67.13 percent vs. 60 percent).^6^ Additionally, there is also a significant difference in the proportion of farmers using chemical pesticides (74.63 percent vs. 70.41 percent). Due to wide variation in reported expenditures, however, the differences in expenditures for these chemical inputs are largely not statistically significant. We do, however, find that farmers in *meso-group 1* have significantly higher expenditures on DAP (BDT 876.56 vs. BDT 667.50) and MoP (BDT 628.95 vs. BDT 554.04), though this latter effect is only marginally significant.

There are also significant differences along some of the financial dimensions. Although members of meso*-*group 2 are less likely to have a savings account with a bank (27.39 percent vs. 30.50 percent), they are significantly more likely to be members of informal savings groups (23.50 percent vs. 16.04 percent). In many ways, given the relatively low penetration of formal financial institutions in rural Bangladesh, these informal savings groups provide a vital service, and may indeed substitute for formal financial risk management solutions like index insurance. We also note that the farmers in meso*-*group 2 have higher savings ability during a ‘good month,’ though we note this difference is only marginally statistically significant. Although this difference may be only marginally significant, the different patterns that are observed in the reported sufficiency of household savings cannot simply be attributed to chance. Farmers in meso*-*group 2 are more likely to report having more than enough savings (48.95 percent vs. 41.79 percent) and are less likely to lack sufficient savings for consumption needs (21.47 percent vs. 23.13 percent). We also observe some differences in the spatial distribution of members of the meso-groups. Our results suggest that more farmers from Bogra Sadar fall into meso-group 1, while more farmers from Gabtoli and Sariakandi upazilas belong to meso-group 2. Bogra Sadar upazila contains the city of Bogra, which is the largest urban area in our study area. Despite being rural, most of the farmers in our sample from Bogra Sadar upazila probably have relatively easy access to financial services, whereas farmers in the other two upazilas might not have easy access to financial or other services that are available in urban areas.

## 5 Discussion

What do these differences across the two meso-groups suggest, particularly in terms of how agronomic and financial risk management interventions might be targeted? In some ways, relatively straightforward narratives emerge. Within each meso-group, the majority of farmers (those belonging to classes AA1 and AA2, respectively) fully attend to each of the attributes, while only a relatively small portion (only 2 percent in meso-group 1 and 19 percent in meso-group 2) demonstrate some level of attribute non-attendance. Recall that farmers in AA1, who constitute the vast majority of meso*-*group 1, derive significant disutility from the drought tolerance yield distribution. The drought-tolerant yield distribution, coincidentally, conferred a modest yield penalty under normal rainfall or well-irrigated conditions (6.5 t/ha vs. 5.2 t/ha) relative to the most commonly cultivated high yielding variety in Bangladesh (BR 11). The fact that farmers from this meso-group also appear more likely to apply phosphatic and potassic fertilizers vis-à-vis those in meso*-*group 2 may indicate that they are ultimately most concerned with attaining the highest possible yields.

While we do not have data to definitively conclude the reasoning behind these preferences, we suggest one possibility is that they discount the likelihood of yield loss due to drought. Consequently, they do not care as much about the potential reduction in yield variability under drought stress that the drought-tolerant varieties might confer so much as the yield penalty that they may experience in the absence of drought stress. This view is supported by the fact that farmers in this meso-group, particularly those from class AA1, have stronger preferences for the limited coverage insurance components than their corresponding full coverage counterparts. Since the limited coverage insurance components are essentially designed to complement the performance of the drought-tolerant variety, they only begin to provide insurance payouts after longer periods without rainfall. This would suggest, or at least support the notion, that farmers in this meso-group expect a lower probability of experiencing a drought relative to what the objective evidence might suggest. An alternative explanation is that this behavior reflects risk preferences, specifically that farmers in meso-group 1 may be systematically less sensitive to risks than farmers in meso-group 2. Previous research has demonstrated that risk preferences affect the way which farmers value different seed attributes (Ward et al., 2014).

Despite their preferences for higher yields and their greater likelihood for applying these chemical fertilizers, farmers in meso-group 1 reported *aman* rice yields from 2013 that were statistically indistinguishable those from meso*-*group 2. They also appear equally likely to use irrigation (72.85 percent vs. 72.5 percent), so more than a quarter of the farmers in this meso*-*group are still potentially exposed to yield losses attributable to droughts. If it is true that these farmers discount the likelihood of yield losses due to droughts, this perception and the psychological emphasis on attainable yields is not so much based upon objective reality as it is on subjective perceptions. As previously indicated, we are not in a position to definitively diagnose the underlying cause. Regardless, the results are suggestive that there is a nontrivial group of farmers who, despite potentially being susceptible to droughts, would not be interested in adopting a drought-tolerant rice variety if there was a sizeable yield penalty under normal conditions vis-à-vis a more familiar variety, other things equal.

When it comes to the second meso-group, we estimate that almost 20 percent ignore the financial attributes incorporated in the choice set, including both the insurance components as well as the bundle price. While the remaining 80 percent do not explicitly ignore these financial attributes, the magnitudes of their preference coefficients are quite low, and their characteristics suggest that they may have adequate financial resources such that formal insurance may not be particularly appealing. The descriptive statistics in Table 3 suggest that the farmers in this meso-group may have adequate means for either self-insuring or accessing informal social safety nets to help them cope with the adverse effects of droughts when they arise. As a result, they may not feel as though they need insurance. Part of this could be due to a limited understanding of the role of insurance in agricultural risk management: Overall, experience with insurance in Bangladesh remains rather limited. Alternatively, these farmers may have had some experience with insurance, either directly or indirectly, but they may have felt as though insurance was inadequate to meet their agricultural risk management needs. It has been often observed with index insurance pilots in many developing countries that basis risk (i.e., the probability that an insured might experience a loss and yet not receive a payment from the insurer) remains a significant constraint to sustained insurance demand. Given that nearly 75 percent of farmers in meso*-*group 2 used irrigation during the most recent *aman* rice season, they may, on average, feel as though their drought risks are effectively managed by irrigation, and insurance may just be an expensive tool that fails to provide any additional risk management. Again, we are unable to make a full diagnosis. But as before, there seems clear evidence that farmers possessing sufficient financial resources and having access to other informal savings and credit facilities may not be especially interested in index insurance products along the lines of the ones described in the present study.

## 6 Concluding remarks

Farmers in Bangladesh, like in other parts of the developing world, face a constant threat of extreme weather events. There are a number of financial and technological innovations that could potentially mitigate weather-related production risks. However, the effectiveness and adoption of those interventions depend on farmers’ preferences and how they process the various information related to them. As such, understanding which innovations are actively evaluated by farmers and which ones are not is important for the development of specific rice varieties and insurance packages that meet the needs of farmers. In this study, we use stated choice data to study farmer preferences for two alternate strategies to manage risks associated with drought in Bangladesh. Utilizing an equality constrained latent class (ECLC) model specification, we capture farmer preference heterogeneity and capture differing information processing strategies regarding DT rice varieties and WII. We identify two overarching preference classes; these meso-groups differ regarding both taste intensity and attribute attendance patterns. We find that farmers belonging to meso*-*group 1 generally dislike or ignore the seed characteristics – most notably the drought-tolerant yield distribution – and those in meso*-*group 2 have relatively weak preferences for the insurance components of a bundled DT-WII product. Observable household characteristics provide insight into preference heterogeneity and various patterns of attribute non-attendance.

Our findings imply the need to target interventions to farmers taking into account their preferences and how they process information. Financial and technological innovations may be perceived by farmers differently depending on a number of factors such as farm-level characteristics, access to savings and credit facilities, among others. Our study also suggests that farmers rely on information processing strategies to simplify choice decisions. Since discrete choice experiments like those employed in this paper are increasingly used to estimate market demand and welfare implications of policy changes, our results suggest that it is important to consider the various information processing strategies that are consciously or subconsciously employed so as to improve the reliability of the resulting welfare estimates. This is particularly important when assessing behavior ex ante as these results will inform project design and lay the foundation for policy development. Given that the majority of methodological studies in this area are typically conducted in developed country settings, it is of paramount importance that researchers and development practitioners be aware of both the limitations and recent developments in the choice experiment literature. Future research should focus on corroborating findings from these methodological contributions in developing country applications, particularly in the areas of agriculture and natural resource management, where much work remains to be done.

## Declaration of Competing Interest

There is no conflict of interest.

## Figures and Tables

**Table A1:** Model information criteria comparison.

Model No.	Model specification	Log-Lik.	No. Par.	BIC/n	AIC/n	AIC3/n
1	MNL	−6559	9	1.145	1.139	1.139
2	RPL	−6404	16	1.120	1.110	1.110
3	LC1 + random choice	−6431	10	1.124	1.117	1.117
4	LC1 + ANA seed	−6407	10	1.119	1.113	1.113
5	LC1 + ANA finance	−6482	10	1.133	1.126	1.126
6	LC1 + ANA seed + ANA financial	−6379	11	1.115	1.108	1.108
7	LC1 + ANA seed + ANA financial + random choice	−6285	12	1.100	1.092	1.092
8	LC2	−6348	19	1.117	1.104	1.104
9	LC2 + ANA seed + ANA financial	no convergence	21	–	–	–
10	LC2 + ANA seed + random choice + ANA financial	−6214	22	1.096	1.082	1.082
11	LC3	−6227	29	1.104	1.085	1.085

## Footnotes

1 We point readers interested in choice modelling methods to Hensher, Rose, and Greene (2015).

2 The three *upazilas* were Bogra, Gabtali, and Sariakandi. Bogra is the name of one of the *upazilas* in our study area as well as the name of the encompassing district.

3 The term *aman* is a general term used to describe varieties of rice grown during the summer monsoon period. This contrasts with *boro* rice, which is grown during the post-monsoon (winter) season.

4 1 Bangladeshi Taka (BDT) = 0.0129 USD at the time of the study.

5 While we have reported class probabilities at the sample level in Table 3, each individual also has a probability of belonging to each of the five groups, based on his or her responses to the five choice tasks. We note that, although our results suggest a 3.3 percent chance that a respondent would belong to Class 3, there were, in fact, no respondents for whom the probability of belonging to Class 3 was higher than the probability of belonging to any other class.

6 An exception to this general finding is that the proportion of farmers using urea is essentially the same in each *meso*-group, but this is likely because virtually everyone in the sample uses urea.
